# Correlation between basal cell adenoma and basal cell adenocarcinoma of the salivary gland: a histomorphological and molecular review of 129 cases

**DOI:** 10.1007/s00428-025-04120-7

**Published:** 2025-05-13

**Authors:** Haruna Yagi, Yoshitaka Utsumi, Yuichiro Tada, Satoshi Baba, Toshihide Iwashita, Kennosuke Karube, Makoto Urano, Toshitaka Nagao, Masato Nakaguro

**Affiliations:** 1 Department of Pathology and Laboratory Medicine, Nagoya University Graduate School of Medicine, 65 Tsurumai-Cho, Showa-Ku, Nagoya, 466-8560 Japan; 2 Department of Anatomic Pathology, Tokyo Medical University, Tokyo, Japan; 3 Department of Head and Neck Oncology and Surgery, International University of Health and Welfare, Mita Hospital, Tokyo, Japan; 4 Department of Diagnostic Pathology, Hamamatsu University School of Medicine Hospital, Hamamatsu, Japan; 5 Department of Regenerative and Infectious Pathology, Hamamatsu University School of Medicine, Hamamatsu, Japan; 6 Department of Diagnostic Pathology, Bantane Hospital, Fujita Health University, Nagoya, Japan

**Keywords:** β-catenin, Basal cell adenocarcinoma, Basal cell adenoma, *CTNNB1*, Salivary gland tumor

## Abstract

Basal cell adenoma (BCA) and basal cell adenocarcinoma (BCAC) are salivary gland tumors with biphasic differentiation, composed of luminal ductal cells and abluminal basal cells with a high nuclear-to-cytoplasmic ratio. While BCA is a relatively common benign tumor, BCAC is a rare malignancy, and its genetic context and relationship with BCA remain unclear. We investigated 93 BCA and 36 BCAC cases to further characterize these two tumor entities from histological and molecular perspectives. BCA/BCAC proliferated in a mixture of tubular, trabecular, solid, cribriform, and membranous patterns. A jigsaw puzzle pattern, peripheral palisading, S100-positive stroma, cystic change, and sclerosis were observed in approximately 50% of the cases. BCAC demonstrated the following malignant features: infiltration to surrounding tissue, tumor necrosis, and increased mitotic activity (81%, 22%, and 22%, respectively). The nuclear expression of β-catenin was frequently observed in both BCA and BCAC (89% and 60%), and *CTNNB1* hotspot mutations were detected in 46% and 48% of BCA and BCAC cases, respectively. Tubular patterns of growth, jigsaw puzzle patterns, peripheral palisading, S100-positive stroma, and cystic changes were more common in β-catenin-positive BCA/BCAC than in β-catenin-negative BCA/BCAC. Among the β-catenin-negative BCA/BCAC cases, one case each harbored *PLAG1* and *MYB* rearrangements. We concluded that β-catenin-positive BCA and BCAC share common histologic and molecular features, and BCAC is considered a malignant counterpart of BCA. β-Catenin-negative BCA/BCAC might include morphological mimickers, which can be genetically classified into other tumor types, including pleomorphic adenoma and adenoid cystic carcinoma.

**Supplementary Information:**

The online version contains supplementary material available at 10.1007/s00428-025-04120-7.

## Introduction

**Fig. 1:**
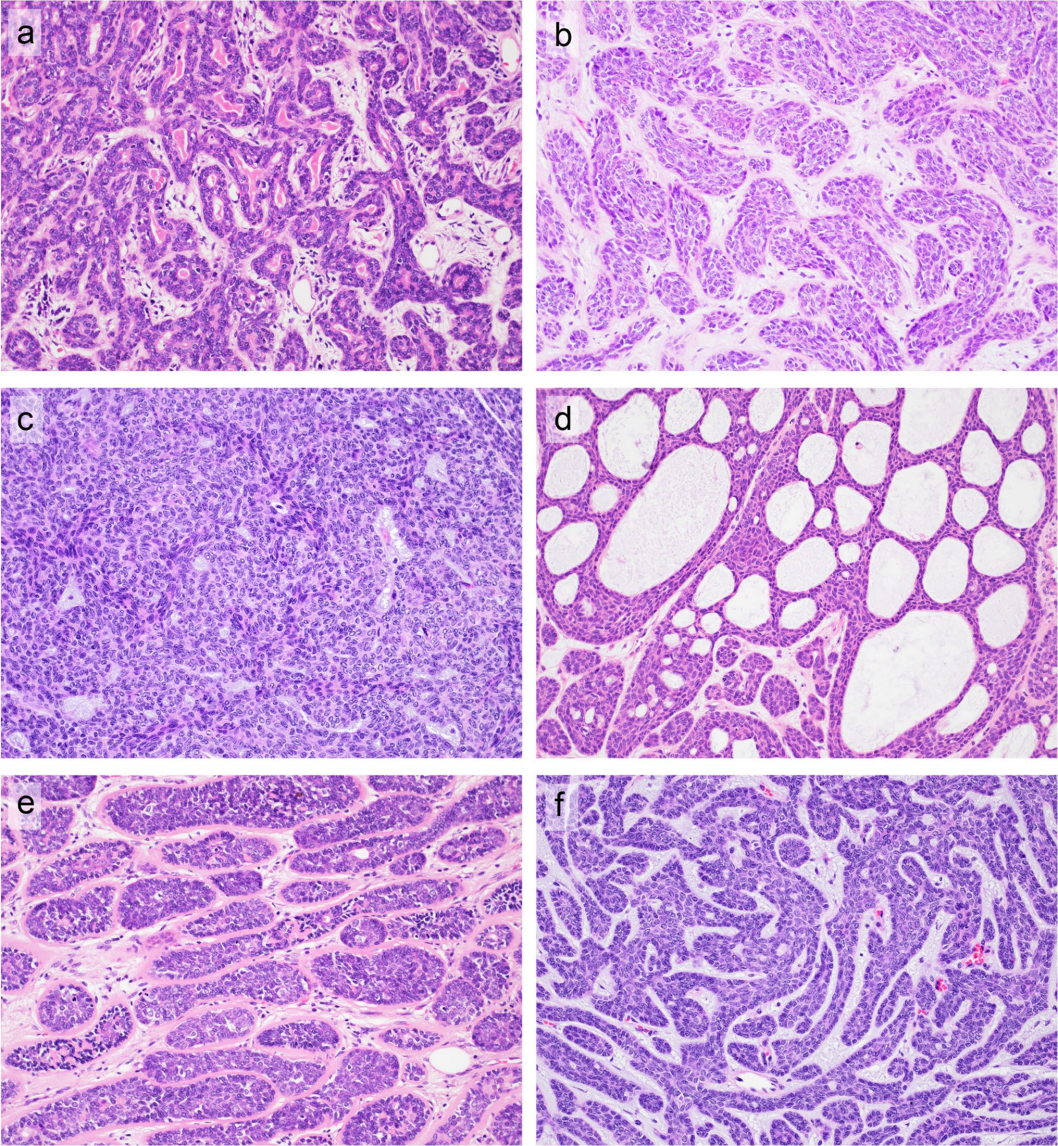

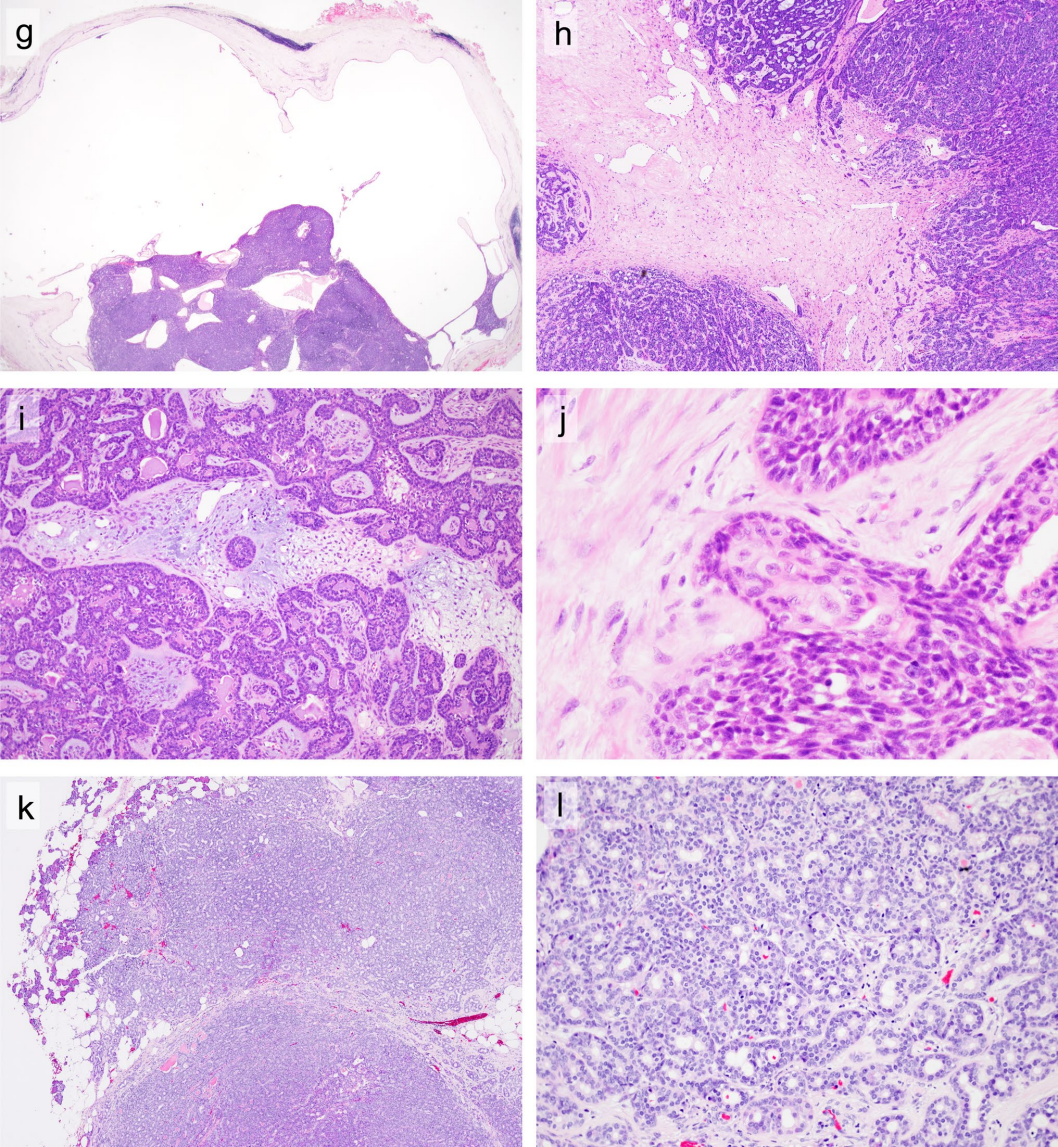
Growth patterns and histologic features of basal cell adenoma (BCA) and basal cell adenocarcinoma (BCAC). The basaloid neoplastic cells of BCA/BCAC proliferate with a combination of several growth patterns (**a**: tubular pattern, **b**: trabecular pattern,** c**: solid pattern, **d**: cribriform pattern, and **e**: membranous pattern). In membranous patterns, the tumor cell nests are surrounded by dense hyaline material. (**f**) Nuclear palisading is observed at the periphery of the tumor cell nests. Tumor cell nests are separated by a narrow stromal component exhibiting a characteristic jigsaw puzzle pattern. Cystic changes (**g**) and sclerosis (**h**) are common features of BCA/BCAC. Myxoid stroma (**i**), squamous differentiation (**j**), and adjacent intercalated duct hyperplasia (IDH) (**k**, **l**) are the less frequently observed histological features. (**k**) Low-power view of the BCA (lower portion) and the adjacent IDH (upper portion). (**l**) High-power view of the IDH. IDH consists of small ducts with a minimal intervening stroma

**Fig. 2:**
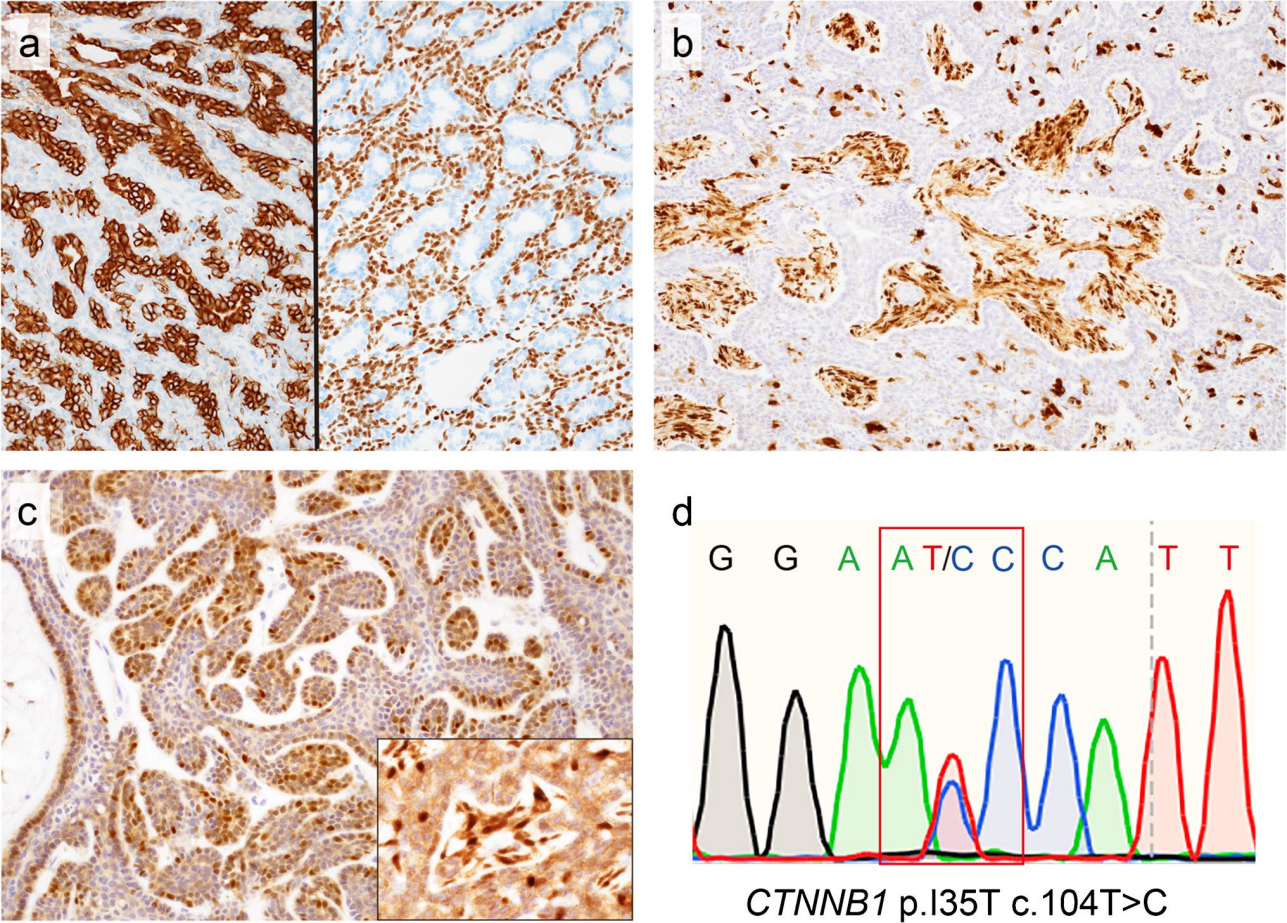
Immunohistochemical and genetic findings of BCA/BCAC. (**a**) Luminal ductal cells are strongly positive for CK7 (left) whereas basal cells are positive for p63 (right). (**b**) S100-positive stromal cells are present. (**c**) The nuclear expression of β-catenin is restricted to a subset of basal cells, while luminal ductal cells are consistently negative for β-catenin. Additionally, some S100-positive spindle stromal cells are positive for β-catenin (inset). (**d**) Sanger sequencing of the *CTNNB1* p.I35T variant

Basal cell adenoma (BCA) and basal cell adenocarcinoma (BCAC) are salivary gland tumors (SGT) characterized by a basaloid appearance due to the high nuclear-to-cytoplasmic ratio of neoplastic cells (Fig. 1). Contrary to the terminology, BCA/BCAC does not consist of a proliferation of basaloid cells alone, but shows biphasic differentiation composed of luminal ductal cells and abluminal cells (Fig. 2a). While BCA is a relatively common benign tumor, accounting for 5–6% of benign SGT, BCAC is a rare malignant SGT [1–3]. BCAC is considered to be a low-grade malignant tumor with a high recurrence rate and rare metastasis, and usually has a good prognosis [4–7]. Although other SGT, such as pleomorphic adenoma (PA), adenoid cystic carcinoma (AdCC), and epithelial-myoepithelial carcinoma (EMC), occasionally exhibit a basaloid appearance (Supplementary Fig. 1), they usually demonstrate, at least in part, their own characteristic histologic features [2, 3, 8–11]. BCA/BCAC are also characterized by specific features, including the jigsaw puzzle pattern, peripheral palisading, and S100-positive stroma [1–3]. BCAC is distinguished from BCA primarily by the presence of infiltration to surrounding tissue. Necrosis and increased mitotic counts are supportive diagnostic features of BCAC according to the 2024 *WHO Classification of Head and Neck Tumours* [1].

BCA has been reported to harbor hotspot point mutations in the *CTNNB1* gene, which encodes β-catenin [6, 12–15]. The mutation inhibits the degradation of β-catenin and activates the Wnt pathway [14]. The detection of nuclear β-catenin accumulation by immunohistochemical staining is another specific feature of BCA and is included in desirable diagnostic criteria in the 2024 *WHO Tumour Classification* [12, 13, 16]. In contrast, in BCAC, the nuclear expression of β-catenin has been described in only a subset of cases, which included one *CTNNB1*-mutated case [5, 6, 8, 13–16]. However, all of these data were from studies with small numbers of cases, and the frequency of the expression of β-catenin and the mutation profile has not been fully elucidated due to the rarity of BCAC.

In this substantial cohort series of BCA and BCAC, we analyzed their histomorphological, immunohistochemical, and genetic characteristics and sought to clarify the relationship between these two entities.

## Materials and methods

### Patients and histological review

This study was approved by the institutional ethics review board of each participating institution (2018–0190), and the need to obtain informed consent was waived due to the retrospective nature of the analysis.

Cases were retrieved from the pathology archives of each participating institution and from consultation cases. The pathology slides were reviewed by four board-certified expert pathologists in this field (H.Y, M.N., M.U., and T.N.) to confirm diagnoses according to the criteria of the *2024 WHO Classification of Head and Neck Tumours* [1]. Histological diagnoses were made based on the histomorphology of hematoxylin and eosin staining; however, when necessary, immunohistochemical staining for various markers was performed. BCAC was distinguished from BCA by the presence of infiltration to the surrounding tissue, necrosis, and/or increased mitoses (> 4 per 2 mm^2^). With reference to the diagnostic criteria of PA, non-neoplastic acini merging at the periphery, capsular invasion, pseudopodia, and multinodular growth were not regarded as diagnostic features of BCAC [1].

In total, 93 BCA and 36 BCAC cases were retrieved for a further analysis. For comparison, we also selected other SGT that at least partially exhibited a basaloid appearance, including 6 PA, 8 AdCC, and 12 EMC cases. The clinical characteristics of PA, AdCC, and EMC are summarized in Supplementary Table 1.

The clinical features (age, sex, anatomical site, and tumor size) of the patients were extracted from the available medical records and pathology reports. We reviewed the pathology slides and evaluated the growth pattern (tubular, trabecular, solid, cribriform, and membranous), capsular invasion or pseudopodia, infiltration into surrounding tissue, perineural invasion, lymphovascular invasion, necrosis, nuclear atypia, mitotic count (> 4 per 2 mm^2^), jigsaw puzzle pattern, peripheral palisading, S100-positive stroma, cystic change, sclerosis, myxoid stroma, squamous differentiation, lipomatous change, and adjacent intercalated duct hyperplasia (IDH). All growth patterns observed in the tumors were counted, even when the pattern was not predominant.

### Immunohistochemistry (IHC)

IHC studies were performed on 4-µm-thick formalin-fixed paraffin-embedded sections. IHC for β-catenin (dilution at 1:150, clone 17 C2, Leica Biosystems, Wetzlar, Germany), S100 (dilution at 1:250, polyclonal, Dako Cytomation, Carpinteria, CA), and Ki-67 (dilution at 1:100, clone MIB-1, Dako Cytomation) was performed for all available cases of BCA and BCAC. IHC for CK7 (dilution at 1:50, clone OV-12/30, Dako Cytomation) and p63 (diluted, clone 4 A4, Nichirei Bioscience, Tokyo, Japan) was performed when necessary. The Ki-67 proliferation (PI) index was assessed in the hotspots.

### Molecular analysis

DNA was extracted from unstained formalin-fixed paraffin-embedded slides using a QIAamp DNA Mini Kit (Qiagen, Valencia, CA, USA). The tumor components on the slides were macrodissected to increase the tumor cell ratio. To detect hotspot mutations in *CTNNB1*, we performed polymerase chain reaction (PCR), followed by Sanger sequencing. The protocol was the same as reported in a previous study [17, 18]. A hotspot mutation analysis of *CTNNB1* (exon 3) was performed for all analyzable cases. Fluorescence in situ hybridization (FISH) for *PLAG1*, *HMGA2*, and *MYB*, as well as Sanger sequencing for *HRAS* (exons 2 and 3) and RAS Q61R IHC [19] were performed for necessary cases. The PCR primers used for Sanger sequencing and the break-apart probes used for FISH studies are summarized in Supplementary Tables 2 and 3.

### Statistical analysis

Fisher’s exact test was used to compare the ratio of non-continuous variables, and Student’s *t*-test was used to compare the average values of continuous variables between the two groups. All statistical analyses were performed using the EZR (Saitama Medical Center, Jichi Medical University, Saitama, Japan) [20]. *p*-values of < 0.05 were considered to indicate statistical significance.

## Results

### Clinical findings

The clinical characteristics of BCA and BCAC cases are summarized in Supplementary Table 4. The mean age of patients with BCA and BCAC was 58.6 and 63.0 years, respectively. The female-to-male ratio was approximately 3:2 for both BCA and BCAC. In BCA, 97% (90 cases) arose in the parotid gland, followed by the parapharyngeal space and submandibular gland. In BCAC, 75% (27 cases) originated from the parotid gland, and the remaining cases arose in the parapharyngeal space, submandibular gland, palate, lip, buccal mucosa, and nasal tract. The mean tumor sizes in BCA and BCAC were 22.2 mm and 23.8 mm, respectively.

### Histological and immunohistochemical findings

The histological findings of BCA and BCAC cases are summarized in Table 1. Most BCA/BCAC cases exhibited multiple growth patterns in each tumor (Fig. 1a–e). Tubular, trabecular, and solid patterns were observed in > 50% of BCA/BCAC tumors, and cribriform and membranous patterns were relatively uncommon. The frequency of each growth pattern did not differ significantly between the BCA and BCAC groups.

**Table 1:** Histologic features of BCA and BCAC, *n* [%]

	BCA	BCAC	*p*-value
**Growth pattern**			
Tubular	65/93 [70.0]	21/36 [58.3]	0.22
Trabecular	73/93 [78.4]	24/36 [66.7]	0.18
Solid	69/93 [74.2]	31/36 [86.1]	0.17
Cribriform	18/93 [19.4]	13/36 [36.1]	0.065
Membranous	8/93 [8.6]	6/36 [16.7]	0.21
**Specific BCA/BCAC features**			
Jigsaw puzzle pattern	53/93 [57.0]	13/36 [36.1]	0.049*
Peripheral palisading	54/93 [58.1]	17/36 [47.2]	0.33
S100-positive stromal cells	70/86 [81.4]	10/24 [41.7]	< 0.01*
**Other features**			
Cystic change	56/93 [60.2]	11/36 [30.6]	< 0.01*
Sclerosis	48/93 [51.6]	18/36 [50.0]	1.0
Myxoid stroma	11/93 [11.8]	2/36 [5.6]	0.51
Squamous differentiation	1/93 [1.1]	3/36 [8.3]	0.066
Lipomatous change	1/93 [1.1]	2/36 [5.6]	0.19
Adjacent intercalated duct hyperplasia (IDH)	5/93 [5.4]	2/36 [5.6]	1.0
**Invasion, nuclear atypia, and proliferation**			
Capsular invasion or pseudopodia	36/93 [38.7]	34/36 [94.4]	< 0.01*
Infiltration to surrounding tissue	0/93 [0]	29/36 [80.6]	< 0.01*
Perineural invasion	0/93 [0]	10/36 [27.8]	< 0.01*
Vascular invasion	0/93 [0]	9/36 [25.0]	< 0.01*
Lymphatic invasion	0/93 [0]	4/36 [11.1]	< 0.01*
Necrosis	2/93 [2.2]	8/36 [22.2]	< 0.01*
Nuclear atypia			
Mild	55/93 [59.1]	3/36 [8.3]	
Moderate	36/93 [38.7]	23/36 [63.9]	
Severe	2/93 [2.2]	10/36 [27.8]	
Mitosis (mean, [range] per 2 mm^2^)	0.41 [0–3]	3.19 [0–20]	< 0.01*
Mitosis (> 4 per 2 mm^2^)	0/93 [0]	8/36 [22.2]	< 0.01*
Ki-67 proliferation index (mean, [range]) (%)	1.93 [0–10]	11.0 [1–40]	< 0.01*
**β-catenin (IHC) and** ***CTNNB1 *hotspot mutation**			
Nuclear expression of β-catenin	79/89 [88.8]	18/30 [60.0]	< 0.01*
Stromal nuclear β-catenin expression in β-catenin (+) cases	48/79 [60.8]	5/18 [27.8]	0.017*
*CTNNB1* hotspot mutation	35/77 [45.5]	12/25 [48.0]	1.0

The jigsaw puzzle pattern (Fig. 1f) and S100-positive stroma (Fig. 2b) were more commonly observed in BCA than in BCAC (57% vs. 36%, *p* = 0.049 and 81% vs. 42%,* p* < 0.01, respectively).

Cystic changes were more frequent in BCA (60%) than in BCAC (31%) (*p* < 0.01) (Fig. 1g). Sclerosis (Fig. 1h) was observed in ≥ 50% of cases in both BCA and BCAC. Myxoid stroma (Fig. 1i), squamous differentiation (Fig. 1j), lipomatous change, and adjacent IDH (Fig. 1k, l) were observed in 1–12% of the BCA/BCAC cases.

**Fig. 3:**
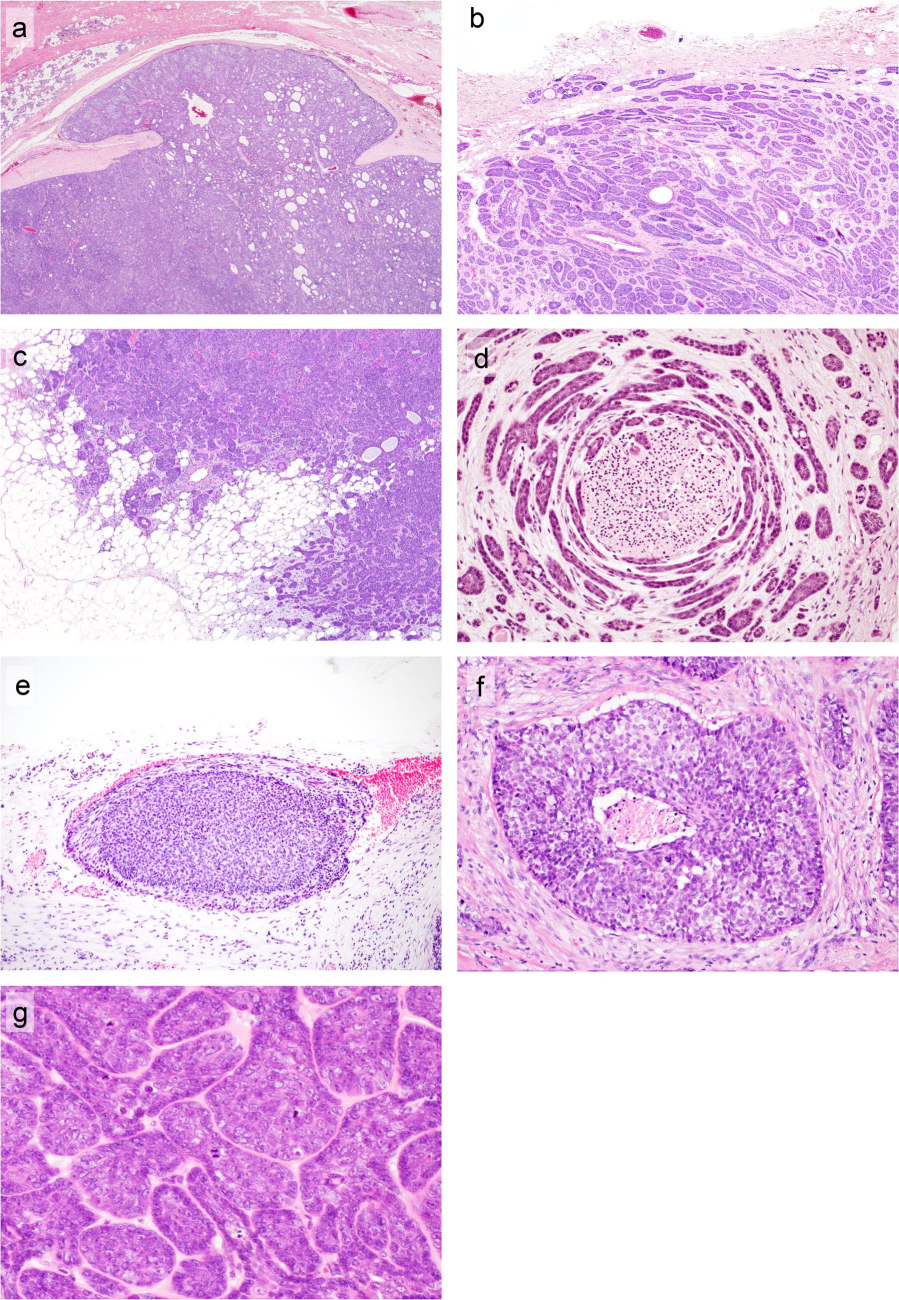
Invasion, infiltration, nuclear atypia, and necrosis in BCA and BCAC. Capsular invasion (**a**) and pseudopodia (**b**) of BCA. These findings are not diagnostic features of a malignancy. **c**–**f** Malignant features of BCAC. **c** Infiltration of the surrounding tissue is the major diagnostic criterion for BCAC. This patient was positive for nuclear β-catenin. **d** Perineural invasion. This patient was positive for nuclear β-catenin and harbored the *CTNNB1* p.I35T variant. **e** Vascular invasion. This patient was negative for nuclear β-catenin. **f** Necrosis. This patient was negative for nuclear β-catenin. **g** Severe nuclear atypia. This patient was positive for nuclear β-catenin and harbored the *CTNNB1* p.I35T variant

Capsular invasion or pseudopodia were present in 39% and 94% of BCA and BCAC cases, respectively (Fig. 3a, b). Infiltration into the surrounding tissue, the major diagnostic criterion for BCAC, was observed in 81% of BCAC cases (Fig. 3c). Perineural, vascular, and lymphatic invasion were found in 28%, 25%, and 11% of BCAC cases, respectively (Fig. 3d, e). Necrosis was noted in 22% of patients with BCAC (Fig. 3f). Necrosis was also identified in 2 BCA cases; however, it was considered a degenerative change caused by preoperative needle biopsy. While severe nuclear atypia was more common in BCAC than in BCA (Fig. 3g), 8% of the BCAC cases showed mild nuclear atypia indistinguishable from BCA. The mean mitotic count was significantly higher in BCAC than in BCA (3.2/2 mm^2^ vs. 0.4/2 mm^2^) (*p* < 0.01). There was a large variation in the mitotic count in the BCAC cases. While a mitotic count > 4/2 mm^2^ was observed in 22% of BCAC cases, the mitotic count in all BCA cases was < 4/2 mm^2^. The Ki-67 PI index also varied greatly in BCAC; however, the mean Ki-67 PI was significantly higher in BCAC than in BCA (11% vs. 1.9%) (*p* < 0.01).

### β-Catenin immunohistochemistry and *CTNNB1* hotspot mutation

The nuclear β-catenin expression in BCA and BCAC is summarized in Table 1 and Supplementary Tables 5 and 6. The nuclear expression of β-catenin was significantly more common in BCA (79/89 cases, 89%) than in BCAC (18/30 cases, 60%) (*p* < 0.01). The nuclear expression of β-catenin was limited to basal cells, whereas luminal ductal cells were consistently negative for β-catenin (Fig. 2c). Among β-catenin (+) cases, the nuclear expression of β-catenin in stromal cells was also observed in 61% of BCA (48/79 cases) and 28% of BCAC cases (5/18 cases) (Fig. 2c inset). β-catenin (+) BCA/BCAC were localized not only in the parotid gland but also in other anatomic sites, including the submandibular gland and nasal tract (Supplementary Table 7). No nuclear β-catenin expression was identified in any of the PA, AdCC, or EMC cases that were examined.

The *CTNNB1* hotspot mutation profiles of BCA/BCAC are summarized in Table 1 and Supplementary Tables 5 and 6. *CTNNB1* hotspot mutations were detected in 46% and 48% of the BCA and BCAC cases, respectively. With the exception of two cases of BCA and one case of BCAC, all *CTNNB1*-mutated BCA/BCAC cases showed the nuclear expression of β-catenin. The majority of hotspot mutations were p.I35 T (Fig. 2d), followed by p.T42P, p.S45 C, and p.G38 del. All PA, AdCC, and EMC cases lacked *CTNNB1* hotspot mutations.

The correlation between the histological features and the nuclear expression of β-catenin in BCA/BCAC is summarized in Table 2 and Supplementary Table 8. Tubular growth, jigsaw puzzle pattern, peripheral palisading, S100-positive stromal cells, and cystic changes were more common in β-catenin (+) BCA/BCAC cases than in β-catenin (−) cases (71% vs. 41%, *p* = 0.011, 57% vs. 23%, *p* < 0.01, 59% vs. 32%, *p* = 0.032, 86% vs. 14%, *p* < 0.01, and 58% vs. 23%, *p* = 0.01, respectively). In contrast, membranous growth pattern and squamous differentiation was more common in β-catenin (−) BCA/BCAC cases than in β-catenin (+) cases (23% vs. 6%, *p* = 0.03, and 14% vs. 1%, *p* = 0.02).

**Table 2:** Comparison of histological features between β-catenin-positive and β-catenin-negative cases of BCA/BCAC, *n* [%]

	β-catenin (+) BCA/BCAC	β-catenin (−) BCA/BCAC	*p*-value
**Growth pattern**			
Tubular	69/97 [71.1]	9/22 [40.9]	0.011*
Trabecular	75/97 [77.3]	13/22 [59.1]	0.11
Solid	76/97 [78.4]	17/22 [77.3]	1.0
Cribriform	23/97 [23.7]	2/22 [9.1]	0.16
Membranous	6/97 [6.2]	5/22 [22.8]	0.030*
**Specific BCA/BCAC features**			
Jigsaw puzzle pattern	55/97 [56.7]	5/22 [22.8]	< 0.01*
Peripheral palisading	57/97 [58.8]	7/22 [31.8]	0.032*
S100-positive stromal cells	76/88 [86.4]	3/21 [14.3]	< 0.01*
**Other features**			
Cystic change	56/97 [57.7]	5/22 [22.8]	0.010*
Sclerosis	51/97 [52.6]	8/22 [36.4]	0.24
Myxoid stroma	8/97 [8.2]	3/22 [13.6]	0.42
Squamous differentiation	1/97 [1.0]	3/22 [13.6]	0.020*
Lipomatous change	3/97 [3.1]	0/22 [0]	1.0
Adjacent intercalated duct hyperplasia (IDH)	4/97 [4.1]	0/22 [0]	1.0

### Molecular analysis of β-catenin-negative cases

We performed further molecular analysis of β-catenin-negative BCA/BCAC cases to exclude the possibility of other tumor types. For the seven β-catenin-negative BCA cases, we considered the possibility of PA and performed *PLAG1* FISH analysis. One BCA case was positive for *PLAG1* break-apart, and this case was considered PA based on molecular findings (Supplementary Fig. 2a). We also performed *HMGA2* FISH for the other six *PLAG1* FISH-negative cases. Four of them were negative for *HMGA2* break-apart, and the other two were not analyzable.

For five β-catenin-negative BCAC cases with highly infiltrative growth and moderate-to-severe nuclear atypia, we performed *MYB* FISH to exclude the possibility of AdCC. One case was positive for *MYB* break-apart and was deemed AdCC (Supplementary Fig.2b). Although the possibility of *MYBL1*-rearranged or rearrangement-negative AdCC could not be excluded, the other four cases were exclusively diagnosed with BCAC based on their morphology.

For two BCA and two BCAC cases, we performed Sanger sequencing for *HRAS* or RAS Q61R IHC to exclude EMC [19]. *HRAS* hotspot mutations and RAS Q61R expression were not detected in any of the cases.

## Discussion

In this study, we examined the histological and genetic features of BCA and BCAC to further characterize these two entities. Although early genetic studies have stated that BCAC lacks *CTNNB1* hotspot mutations and may arise from a distinct mechanism from BCA [6, 13], BCAC cases with *CTNNB1* hotspot mutations have been recently demonstrated [14, 15]. However, since previous analyses of BCAC were limited to single case reports or small case series studies, we performed both β-catenin IHC and *CTNNB1* Sanger sequencing on the largest number of BCAC cases, along with BCA cases. This study revealed that a comparable percentage of BCAC cases harbored *CTNNB1* hotspot mutations as BCA cases (BCA, 46%; BCAC, 48%). These results suggest that BCA and BCAC share a common pathogenic pathway, at least in part.

On the other hand, the nuclear expression of β-catenin was more prevalent than *CTNNB1* hotspot mutations, and it was more common in BCA than in BCAC (89% vs. 60%, *p* < 0.01). These results reflected the low prevalence of β-catenin (+)/*CTNNB1* wild-type tumors in BCAC (Supplementary Table 5). The mechanism underlying the nuclear expression of β-catenin in *CTNNB1*-wild type BCA/BCAC is still unknown. The driver genes of these tumors may be *CYLD* or other Wnt pathway genes. The pathological behavior of these tumors might differ from *CTNNB1*-driven tumors. Considering the low prevalence of β-catenin (+)/*CTNNB1* wild-type BCAC, these tumors might tend to be more benign, or, in other words, non-invasive, than *CTNNB1*-driven basal cell neoplasms. *CYLD* gene alterations have been observed in a subset of BCA and BCAC [21, 22]. This gene alteration is associated with Brooke-Spiegler syndrome, which is characterized by multiple cutaneous cylindromas, spiradenomas, and membranous BCA in the parotid gland [23–25]. Inhibition of *CYLD* promotes Wnt-induced β-catenin stabilization and may cause the nuclear expression of β-catenin [3, 21]. No patients with Brooke-Spiegler syndrome were included in the present study. *CYLD* mutation was found in membranous BCA but not in conventional BCA in a previous study [13]. A membranous pattern was observed in limited foci of 9% of BCA cases and 17% of BCAC cases (Table 1), and it was the predominant feature in six BCA cases. Three of these six cases showed nuclear expression of β-catenin associated with a *CTNNB1* hotspot mutation. *CYLD* alterations, including mutations and deletions, are beyond the scope of this study. Other Wnt pathway genes include *APC* and *AXIN1.* The reported frequency of mutations in these genes is very low [14].

In the present study, specific histological findings of BCA/BCAC, including a jigsaw puzzle pattern, peripheral palisading, and S100-positive stroma, were significantly more common in nuclear β-catenin (+) BCA/BCAC cases than in nuclear β-catenin (−) cases. These results suggest that although β-catenin (+) BCA/BCAC and β-catenin (−) BCA/BCAC share some common histological features and are classified as the same tumor entity, β-catenin (+) BCA/BCAC may be a distinct subtype with characteristic histology and oncogenic mechanisms. The current WHO definitions of BCA (a benign biphasic salivary gland neoplasm composed of basaloid and luminal cells) and BCAC (a malignant infiltrative basaloid salivary gland tumor composed of a mixture of basal and ductal cells) remain valid [1]; however, it is of note that some findings, including peripheral palisading, may not necessarily be observed in all cases, particularly when the tumors are negative for β-catenin.

The nuclear expression of β-catenin was limited to basal cells and S100-positive stromal cells, whereas the luminal ductal cells were consistently negative. The nuclear expression of β-catenin in S100-positive stromal cells suggested that they were neoplastic cells but not reactive mesenchymal cells [13, 26, 27]. The diagram of Sanger sequencing showed almost equal height in the original and mutated bases (Fig. 2d). This result suggested that both basal cells and luminal cells had mutated alleles. Since the cellular functions of luminal ductal cells and basal cells might be different, the expression of proteins differs between these two cell types. Based on these results, basal cells might be more dependent on the Wnt-signaling pathway than luminal ductal cells. A similar basal cell (or myoepithelial cell)-specific protein expression has been reported in AdCC (MYB immunohistochemistry), EMC (RAS Q61R immunohistochemistry), and PA (PLAG1 and HMGA2 immunohistochemistry) [19, 28–30].

BCAC is distinguished from BCA by an infiltration to surrounding tissue, necrosis, and/or increased mitotic activity [1]. In the current study, these features were observed in 81%, 22%, and 22% of BCAC cases, respectively (Table 1). Other features suggestive of malignancy, including perineural, vascular, and lymphatic invasion, were observed in 11–28% of BCAC cases. On the other hand, BCA cases lacked these features. The definition of infiltration was ambiguous in the *2024 WHO Classification*. We adapted the diagnostic criteria of PA, where capsular invasion, pseudopodia, and multinodular growth were not regarded as indicators of malignancy [1, 9]. One previous report compared the clinicopathological characteristics of BCA with and without capsular invasion [5]. The results revealed that although BCA with capsular invasion was larger in size and proliferated with a cribriform pattern more frequently than BCA without capsular invasion, tumor recurrence was not detected in either group. In the present study, 39% of BCA cases exhibited capsular invasion or pseudopodia, and the Ki-67 labeling index of BCA cases with capsular invasion was slightly higher than that of BCA without capsular invasion, but the difference was not statistically significant (2.21 vs. 1.77, *p* = 0.29, data not shown). A recent study revealed that BCAC exhibits a methylation profile distinct from that of BCA [31]. While the result of methylation analyses supported the histologic diagnosis, it should be borne in mind that the difference might have been caused by the presence of a *CTNNB1* mutation, as *CTNNB1*-mutated BCAC cases were not included in the study [31].

Considering the presence of common genetic alterations between BCA and BCAC, there should be BCAC cases arising from pre-existing BCA. However, because the cytological features of BCAC are similar to those of BCA [1], separation of the BCAC component from the BCA component is usually difficult. In our cohort, 72% of the BCAC cases exhibited mild to moderate nuclear atypia. Although there have been reports of carcinoma arising from BCA, the carcinoma components were not necessarily BCAC, but salivary duct carcinoma or adenocarcinoma, NOS, were included [4, 7]. No BCAC arose in the pre-existing BCA in the current cohort.

Other rare histologic features included the presence of IDH, which is a benign proliferation of bilayered intercalated duct and included in the *2024 WHO Classification* as a benign tumor designated intercalated duct hyperplasia and adenoma [1, 32–34]. Since a subset of IDH has been reported to coexist with BCA or EMC and to carry *CTNNB1* or *HRAS* hotspot mutations, IDH was suggested to be a precursor lesion of BCA or EMC [34]. In the present study, all cases (4/4) with adjacent IDH showed the nuclear expression of β-catenin in the BCA component, and three of four cases also showed the nuclear expression of β-catenin in the IDH component. Lipomatous stroma was observed in three cases, which corresponded to tumors designated as basal cell lipoadenomas in a previous study [35].

The diagnosis of biphasic SGT is challenging. PA, AdCC, and EMC occasionally show basaloid morphology and mimic BCA/BCAC [2, 8–11]. Although we reviewed all tumor slides and excluded other differential diagnoses, from a molecular perspective, one case each of BCA and BCAC should have been classified as PA and AdCC, respectively. These results suggest that there may be cases of other tumor types that cannot be differentiated from BCA/BCAC by histomorphology alone. Although the nuclear expression of β-catenin was rarely reported in AdCC or EMC, all of the examined PA, AdCC, and EMC cases were negative for nuclear β-catenin in this study [13, 14, 36].

This study had two major limitations. First, we did not analyze the prognosis or follow-up data. Because BCAC is extremely rare, and the majority of BCAC cases were obtained from consultation files, follow-up data were not available in the present study. To evaluate the biological behavior of BCAC, more cases with full clinical information are necessary. Second, the genetic analyses were mostly limited to *CTNNB1*. Comprehensive genome profiling of Wnt pathway genes and *CYLD* is warranted to elucidate the mechanism of β-catenin (+) *CTNNB1*-wild type BCA/BCAC. Comprehensive genome profiling is also useful for investigating the oncogenic pathways of BCAC by comparing the molecular profiles of BCA and BCAC.

In conclusion, BCA and BCAC share common histologic features and *CTNNB1* hotspot mutations, and BCAC is considered a malignant counterpart of BCA. Specific BCA/BCAC features are more commonly observed in nuclear β-catenin (+) BCA/BCAC cases than in nuclear β-catenin (−) BCA/BCAC cases. The nuclear expression of β-catenin and *CTNNB1* mutations is specific to BCA/BCAC.

## Supplementary Information

Below is the link to the electronic supplementary material.

ESM 1 (PDF 105 KB)

ESM 2 (PDF 71.6 MB)

ESM 3 (PDF 19.8 MB)

## Data Availability

The datasets used and/or analyzed during the current study are available from the corresponding author on reasonable request.
