# Supplementary material for: Correlation between basal cell adenoma and basal cell adenocarcinoma of the salivary gland: a histomorphological and molecular review of 129 cases
Source: Virchows Arch. 2025 May 13;487(1):75–86. doi: 10.1007/s00428-025-04120-7 (PMC12289828; doi:10.1007/s00428-025-04120-7)
Supplement: Supplementary file 1 — (PDF 105 KB) [file 428_2025_4120_MOESM1_ESM.pdf]

Supplementary Table 1. Cilinical findings of salivary gland tumors resembling BCA or BCAC

| | PA (n=6) | AdCC (n=8) | EMC (n=12) |
|---|---|---|---|
| Age (mean [ranege]) (y) | 71.0 [43-87] | 62.0 [37-82] | 70.1 [49-89] |
| Sex (n [%]) | | | |
| Female | 2 [33.3] | 1 [12.5] | 4 [33.3] |
| Male | 4 [66.7] | 7 [87.5] | 7 [58.3] |
| Unknown | 0 [0] | 0 [0] | 1 [8.3] |
| Anatomic site (n [%]) | | | |
| Parotid gland | 3 [50.0] | 4 [50.0] | 10 [83.3] |
| Submandibular gland | 0 [0] | 2 [25.0] | 1 [8.3] |
| Sublingual gland | 0 [0] | 1 [12.5] | 0 [0] |
| Palate | 1 [16.7] | 0 [0] | 0 [0] |
| Maxilla | 1 [16.7] | 1 [12.5] | 0 [0] |
| Unknown | 1 [16.7] | 0 [0] | 1 [8.3] |
| | | | |
| Nuclear expression of β-catenin | 0/6 | 0/7 | 0/2 |
| *CTNNB1* hotspot mutation | 0/6 | 0/7 | 0/10 |

PA: pleomorphic adenoma, AdCC: adenoid cystic carcinoma, EMC: epithelial-myoepithelial carcinoma

Supplementary Table 2. PCR primers used for sanger sequencing

| Gene | Direction | Sequence (5' to 3') |
|---|---|---|
| *CTNNB1* | Forward | TTTGATGGAGTTGGACATGG |
| | Reverse | AAAATCCCTGTTCCCACTCA |
| *HRAS* exon2 | Forward | CAGGCCCCTGAGGAGCGATG |
| | Reverse | TTCGTCCACAAAATGGTTCT |
| *HRAS* exon3 | Forward | TCCTGCAGGATTCCTACCGG |
| | Reverse | GGTTCACCTGTACTGGTGGA |

Supplementary Table 3. Probes used for break apart fluorescence in situ hybridization (FISH)

| Gene | Probes |
| --- | --- |
| *PLAG1* | PLAG1 Break-Apart FISH Probe Kit (Agilent Technologies, Santa Clara, CA) |
| *HMGA2* | HMGA2 Break Apart FISH Probe (Guangzhou LBP Medicine Science and Technology, China) |
| *MYB* | ZytoLight SPEC MYB Dual Color Break Apart Probe (Zytovision, Bremerhaven, Germany) |

Supplementary Table 4. Clinical characteristics of patients with BCA and BCAC

| | BCA (n=93) | BCAC (n=36) |
|---|---|---|
| Age (mean [range]) (y) | 58.6 [24-84] | 63.0 [24-86] |
| Sex (n [%]) | | |
| Female | 54 [58.7] | 22 [64.7] |
| Male | 38 [41.3] | 12 [35.3] |
| Anatomic site (n [%]) | | |
| Parotid gland | 90 [96.8] | 27 [75.0] |
| Parapharyngeal space | 2 [2.2] | 1 [2.8] |
| Submandibular gland | 1 [1.1] | 3 [8.3] |
| Palate | 0 [0] | 2 [5.6] |
| Lip | 0 [0] | 1 [2.8] |
| Buccal mucosa | 0 [0] | 1 [2.8] |
| Nasal tract | 0 [0] | 1 [2.8] |
| Size (mean [range]) (mm) | 22.2 [5-50] | 23.8 [10-55] |

Supplementary Table 5. Nuclear expression of β-catenin and *CTNNB1* mutation analysis, n [%]

| Nuclear expression of β-catenin / *CTNNB1* mutation | BCA (n=74) | BCAC (n=25) |
|---|---|---|
| (+)/(+) | 31 [41.9] | 11 [44.0] |
| (+)/(-) | 35 [47.3] | 5 [20.0] |
| (-)/(+) | 2 [2.7] | 1 [4.0] |
| (-)/(-) | 6 [8.1] | 8 [32.0] |

Supplementary Table 6. *CTNNB1* mutation status in BCA and BCAC, n [%]

| | BCA (n=77) | BCAC (n=25) |
|---|---|---|
| Wild-type | 42 [54.5] | 13 [52.0] |
| p.I35T | 34 [44.2] | 10 [40.0] |
| p.S45C | 1 [1.3] | 0 [0] |
| p.T42P | 0 [0] | 1 [4.0] |
| p.G38del | 0 [0] | 1 [4.0] |

Supplementary Table 7. Number of β-catenin positive BCA/BCAC cases in each organ, n

| | BCA | BCAC |
|---|---|---|
| Anatomic site | | |
| Parotid gland | 76/90 | 14/27 |
| Parapharyngeal space | 2/2 | 1/1 |
| Submandibular gland | 1/1 | 2/2 |
| Palate | | 1/2 |
| Lip | | 0/1 |
| Buccal mucosa | | |
| Nasal tract | | 0/1 |

Supplementary Table 8. Comparison of histological features between β-catenin positive and negative cases in BCA and BCAC, n [%]

| | BCA | | | BCAC | | |
|---|---|---|---|---|---|---|
| | β-catenin (+) | β-catenin (-) | *p* -value | β-catenin (+) | β-catenin (-) | *p* -value |
| **Growth pattern** | | | | | | |
| Tubular | 55/79 [69.6] | 6/10 [60.0] | 0.72 | 14/18 [77.8] | 3/12 [25.0] | <0.01* |
| Trabecular | 61/79 [77.2] | 8/10 [80.0] | 1.0 | 14/18 [77.8] | 5/12 [41.7] | 0.063 |
| Solid | 60/79 [75.9] | 5/10 [50.0] | 0.13 | 16/18 [88.9] | 12/12 [100] | 0.50 |
| Cribriform | 15/79 [19.0] | 1/10 [10.0] | 0.68 | 8/18 [44.4] | 1/12 [8.3] | 0.049* |
| Membranous | 4/79 [5.1] | 4/10 [40.0] | <0.01* | 2/18 [11.1] | 1/12 [8.3] | 1.0 |
| **Specific BCA/BCAC features** | | | | | | |
| Jigsaw puzzle pattern | 46/79 [58.2] | 4/10 [40.0] | 0.32 | 9/18 [50.0] | 1/12 [8.3] | 0.024* |
| Peripheral palisading | 47/79 [59.5] | 4/10 [40.0] | 0.31 | 10/18 [55.6] | 3/12 [25.0] | 0.14 |
| S100-positive stromal cells | 66/75 [88.0] | 3/10 [30.0] | <0.01* | 10/13 [76.9] | 0/11 [0] | <0.01* |
| **Other features** | | | | | | |
| Cystic change | 50/79 [63.3] | 3/10 [30.0] | 0.083 | 6/18 [33.3] | 2/12 [16.7] | 0.42 |
| Sclerosis | 41/79 [51.9] | 3/10 [30.0] | 0.31 | 10/18 [55.6] | 5/12 [41.7] | 0.71 |
| Myxoid stroma | 8/79 [10.1] | 3/10 [30.0] | 0.10 | 0/18 [0] | 0/12 [0] | 1.0 |
| Squamous differentiation | 0/79 [0] | 1/10 [10.0] | 0.11 | 1/18 [5.6] | 2/12 [16.7] | 0.55 |
| Lipomatous change | 1/79 [1.3] | 0/10 [0] | 1.0 | 2/18 [11.1] | 0/12 [0] | 0.50 |
| Adjacent intercalated duct hyperplasia (IDH) | 4/79 [5.1] | 0/10 [0] | 1.0 | 0/18 [0] | 0/12 [0] | 1.0 |
