# Supplementary material for: Correlation between basal cell adenoma and basal cell adenocarcinoma of the salivary gland: a histomorphological and molecular review of 129 cases
Source: Virchows Arch. 2025 May 13;487(1):75–86. doi: 10.1007/s00428-025-04120-7 (PMC12289828; doi:10.1007/s00428-025-04120-7)
Supplement: Supplementary file 2 — (PDF 71.6 MB) [file 428_2025_4120_MOESM2_ESM.pdf]

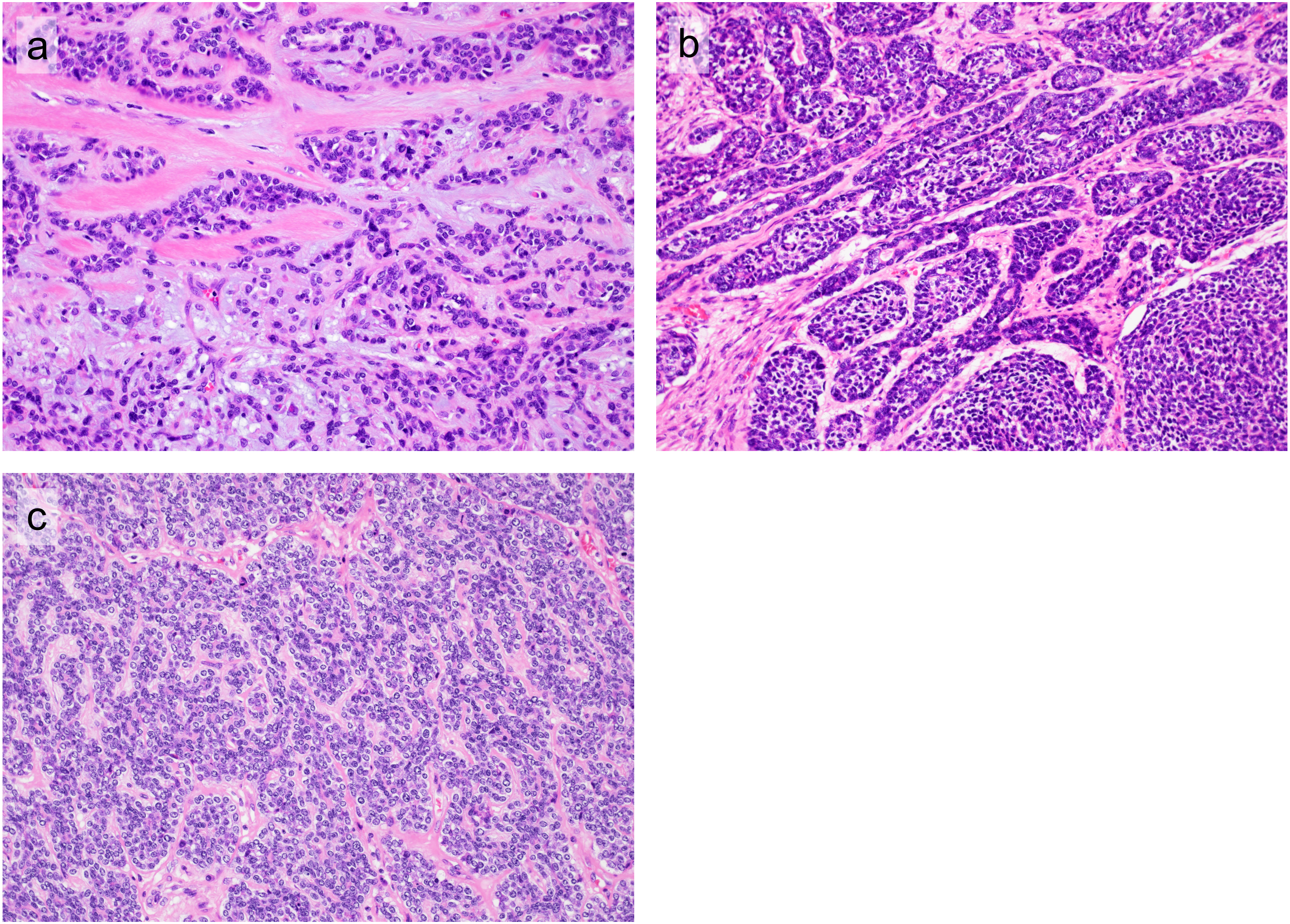

Supplementary Figure 1: Histological features of other tumor entities with a basaloid appearance

(a) Pleomorphic adenoma. (b) Adenoid cystic carcinoma. (c) Epithelial-myoepithelial carcinoma.
