# Supplementary material for: Correlation between basal cell adenoma and basal cell adenocarcinoma of the salivary gland: a histomorphological and molecular review of 129 cases
Source: Virchows Arch. 2025 May 13;487(1):75–86. doi: 10.1007/s00428-025-04120-7 (PMC12289828; doi:10.1007/s00428-025-04120-7)
Supplement: Supplementary file 3 — (PDF 19.8 MB) [file 428_2025_4120_MOESM3_ESM.pdf]

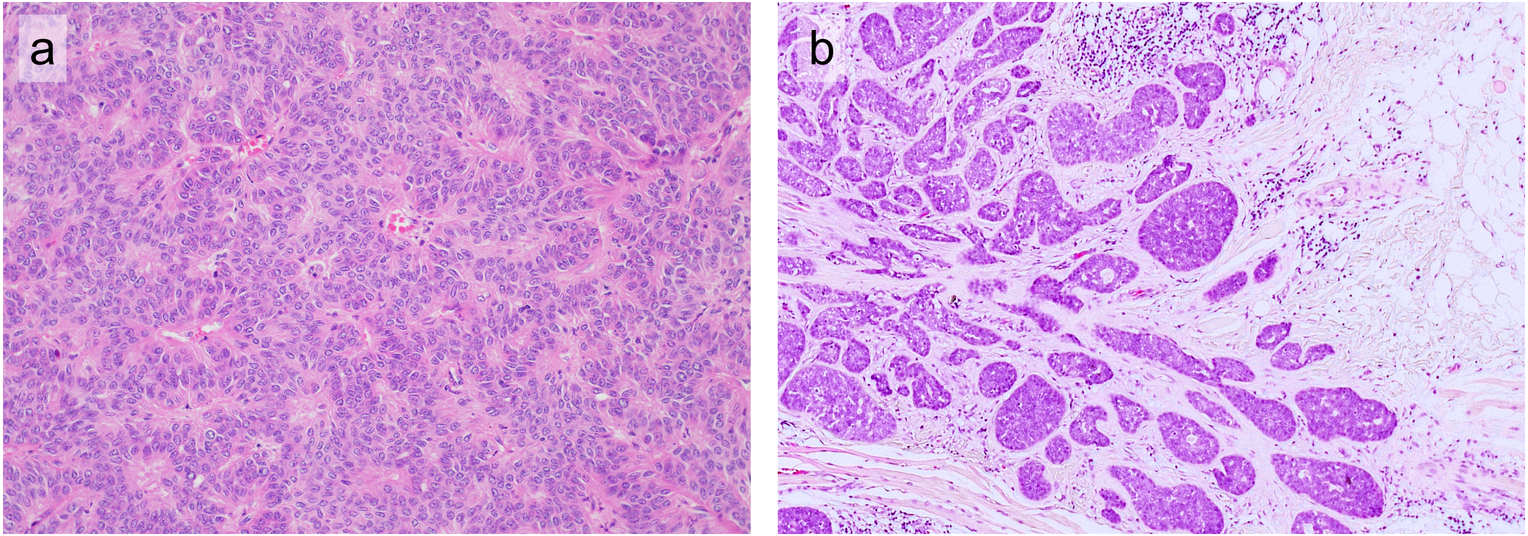

Supplementary Figure 2: Cases of other tumor entities originally diagnosed as “BCA/BCAC” based on the morphology.

(a) This “BCA” case is positive for *PLAG1* rearrangement and is considered to be pleomorphic adenoma from a molecular perspective. (b) This “BCAC” case is positive for *MYB* rearrangement and is deemed to be an adenoid cystic carcinoma.
